# The Work behind Weight-Loss Surgery: A Qualitative Analysis of Food Intake after the First Two Years Post-Op

**DOI:** 10.1155/2014/427062

**Published:** 2014-01-09

**Authors:** Angela A. Geraci, Ardith Brunt, Cindy Marihart

**Affiliations:** Department of Health Nutrition and Exercise Sciences, North Dakota State University, 351 EML, Box 6050, Fargo, ND 58108-6050, USA

## Abstract

*Purpose*. Obesity has reached epidemic proportions in the U.S. and has nearly doubled worldwide since 1980. Bariatric surgery is on the rise, but little focus has been placed on the psychosocial impacts of surgery. The purpose of this study was to explore experiences of patients who have undergone bariatric surgery at least two years before to gain an understanding of the successes and challenges they have faced since surgery. *Methods*. This study used a phenomenological approach, to investigate the meaning and essence of bariatric patients with food after surgery. Semi-structured interviews were conducted on a sample of nine participants who had undergone surgery at least two years prior. *Findings*. Two main themes regarding food intake emerged from the data: (a) food after the first year post-surgery and (b) bariatric surgery is not a magic pill. Upon further analysis, food after the first year post-surgery had four subthemes emerge: diet adherence after the first year post-surgery, food intolerances, amount of food, and tendencies toward coping with food do not magically disappear. *Conclusion*. Findings revealed that post-operative diet and exercise adherence becomes increasingly difficult as weight loss slows. Many participants find that only after the first year after surgery the work really begins.

## 1. Introduction/Purpose

Obesity is now a global public health issue and has nearly doubled worldwide since 1980 [1]. Global obesity estimates from 2008 reported that 35% of adults over the age of 20 were overweight and 11% were considered obese [1]. In addition, obesity rates continue to rise and remain epidemic in the United Sates, as well. More than two-thirds of American adults (68.8%) are considered overweight or obese and more than one-third (35.7%) of adults are obese, constituting more than 78 million American men and women [2]. Individuals who are considered morbidly, or clinically severely, obese (those with BMI of 35 in addition to documented health problems or those with a BMI over 40) are considered potential candidates for bariatric surgery [3].

Individuals elect to undergo bariatric surgery hoping to achieve long-term weight loss and to improve obesity-related comorbid conditions [4]. In 2008, nearly 350,000 bariatric procedures were performed globally [5], with an estimated 220,000 in the United States alone [6].

However, questions still arise on long-term success and its effect on weight loss maintenance and prevention of the return of lifestyle diseases [4, 7]. Previous research indicated that, about a year and a half to two years after bariatric surgery, weight loss from surgery stops and a significant number of individuals begin to regain the weight they had lost [8]. This is when support is needed most; nevertheless, many longitudinal follow-up bariatric surgery studies are short-termed and incomplete [4].

Bariatric surgery is a life-changing procedure that requires a lifelong commitment from the individual. Most research on bariatric surgery primarily focuses on health-related outcomes. Limited research exists examining personal and psychosocial experiences, including satisfaction with weight loss and challenges patients experience following the first year after bariatric surgery. Using a qualitative approach to explore the meaning of the bariatric experience and sharing it with others provides an opportunity for future patients, families of patients, and healthcare providers to gain an understanding of what it is like to undergo a bariatric procedure and to live with the persistent change that accompanies it, after the first two years post-surgery and beyond. This study sought to answer the question: what is the experience of bariatric patients who are at least two years post-surgery?

## 2. Participants and Methods

This study used a qualitative research design, guided by a phenomenological approach, to investigate and describe the meaning and essence of experience of patients at least two years after bariatric surgery [9, 10]. Phenomenological studies are primarily open-ended, searching for themes of meaning in participants' lives [10]. Research is conducted through in-depth interviews. The researcher seeks to understand the deep meaning of a person's experience and how one describes this experience [11]. Exploring the lived experience of patients at least two years after bariatric surgery provided a deeper understanding and awareness of the impact of weight-loss surgery. Through the use of interview questions as a guide, semistructured interviews were conducted (Table 1).

### 2.1. Participants

The method of in-depth, phenomenological interviews applied to a sample of participants who have all experienced similar social conditions gives power to the stories of relatively few participants [10], such as life experience. The researchers used purposeful convenience sampling, which enabled relatively easy access to participants [9]. By contacting participants who were acquainted with the researchers and met inclusion criteria for this study, the researcher was able to conduct semistructured interviews with nine bariatric surgery patients at least two years post-surgery. Criteria for inclusion was set so that the participants who were at least two years after operation and over the age of 18 were recruited through email/telephone regardless of gender, age, surgery type, ethnicity, or socioeconomic status.

The study sample included nine women who ranged from 27 to 57 years of age (*M* = 42). Seven participants had the vertical sleeve gastrectomy (VSG) surgery and 2 had the Roux en Y gastric bypass (RNYB). Self-identified ethnicities of participants were Caucasian (*n* = 5), Hispanic (*n* = 2), and African-American (*n* = 2). Participants ranged from two to seven years after operation (*M* = 3) (Table 2). Weight before surgery following the preoperative diet ranged from 199 pounds to 298 pounds (*M* = 247.4). Initial weight loss after surgery ranged from 58 pounds to 116 pounds (*M* = 88). Weight gain after reaching plateau ranged from none to 48 pounds (*M* = 8.7) (Table 3).

Data analysis included the use of field notes, audiotapes, a reflexive journal, and peer debriefing. Each audiotaped interview was transcribed verbatim and field notes were taken during each interview. After transcription, each interview was printed for clarity. Post-it notes of various colors aided in identifying themes. The first transcript reading helped develop the coding categories, and then the second reading was conducted to start formal coding in a systematic way using colored post-it notes to group related data [9]. The second transcript aided in identifying new categories of information and the two lists were merged into one list, which then represented the final codebook. The transcript of the third interview was compared to the previous code list to see if new categories emerged and this process continued comparing each subsequent transcript for coding categories. Themes were eventually developed into a written description of the participants' experience with bariatric surgery to answer the research question. Quotes from the participants provided a rich, thick description of their experience [11].

### 2.2. Reflexive Researchers' Statement

The researchers personal experience as bariatric patients included preconceived beliefs that bariatric surgery should be for obese individuals who had failed attempts at losing weight through traditional methods with diet and exercise and who need an additional weight-loss tool to aid in achieving a healthy body weight. Being part of the weight-loss surgery community comes with a very “pro-surgery” mentality that the researchers acknowledged. To focus on the participants' experience after bariatric surgery, the researchers were required to bracket those beliefs. Hays and Wood [10] define bracketing as setting aside any assumptions made in everyday life and expressed the need for the researcher to reserve all prejudgments of their experience and rely on intuition and imagination to obtain the picture of the experience.

### 2.3. Trustworthiness

Establishing trustworthiness of the findings was utilized by engaging in peer debriefing of instrument protocol and through prolonged discussions of the research project with peers. Researcher reflexivity was engaged by keeping a journal and field notes, and simultaneous data collection and analysis, which involved collecting and analyzing data simultaneously [9].

## 3. Findings

Review of the data, including individual interviews and field notes, was conducted to analyze and identify themes of the participants' experience with food after bariatric surgery. Two main themes emerged from the data: (a) food after the first year post-op and (b) bariatric surgery is not a magic pill.

### 3.1. Food after the First Year Post-Op

The overarching theme of viewing weight-loss as work after the first year emerged from the data as participants described the mounting difficulty of adhering to the recommended post-op diet once the first year had passed. In addition, tendencies towards using food as comfort and emotional eating were still a struggle that many participants experienced.

…I know I have to keep staying on top of it, it's just that I'm sick of protein, I'm sick of water, I'm sick of working out, I'm just sick of it. I just want eat normal sometimes, but I know I just need to suck it up and deal with it. Finding that balance after the first year…it's so important. [P7]

#### 3.1.1. Diet Adherence after the First Year Post-Op

The recommended diet after bariatric surgery varies among surgeons and across different bariatric surgeries, but there are some commonalities that all participants experienced. Generally agreed upon nutritional guidelines are that supplemental minerals and vitamins should be taken daily, small bites of food should be chewed thoroughly before swallowing, liquids should be either ingested well before meals or at least 30 minutes * *afterwards, and at least 60 grams of proteins should be preferentially eaten before fats and carbohydrates [12–15].

Consuming the right food and the vitamins and doing the right thing with food exist on a continuum…it's not a matter of being all right or all wrong or all good or all bad…every day you're striving to make the best choices you can in that moment. [P3]

In the second year, I noticed I had to be very careful about what I ate and I had to really focus on my vitamins every day and make sure to get my protein and water in every day…the first year, you did not really have to think much and you'd still lose weight. Now I have to focus and pay attention to what I put in my mouth… [P9]

I am more aware of what I'm eating now because my intake is so small, you really have to pay attention to what you're putting in your body. It's quality now, I'm going for the filet minion [sic] before the ground hamburger. [P7]

#### 3.1.2. Food Intolerances

Potential long-term dietary complications include dumping syndrome (e.g., sweating, weakness, hypoglycemia, vomiting, diarrhea, and nausea) related to eating foods high in concentrated sugar or fat [16, 17]. All participants expressed problems with dumping syndrome and/or intolerances to various foods, namely, refined carbohydrates and sugar. It should be noted that food intolerances vary with surgery type. Most participants talked about food intolerances, especially to sugar.

I think the one good thing is that I cannot eat a huge amount of sweets without being nauseated…I can eat a little that's more cracker-ish or cookies…but, like, big bakery pastries or pies, or a big hunk of cake or something, well, that's not going to work very well so I try to avoid it. [P1]

Besides sweets, I have problems with all white carbs like rice, bread, and pasta. It makes me feel woozy. [P9]

#### 3.1.3. Amount of Food

All participants discussed the amount of food they were able to consume in some way, noting reduced intake even years after surgery compared to presurgery amounts.

As far as eating…I can eat many more calories at one time than I used to be able to eat when recently post-op. [P3]

You do have more hunger, though you still cannot eat like you used to…and I have a bit more of a desire to eat than I did the first year…I'm still restricted in how much I can eat and eat about 3 ounces of dense protein and 1 ounce of vegetables and 1 ounce of starch for a meal. [P6]

#### 3.1.4. Tendencies towards Coping with Food Do Not Just Magically Disappear

Many participants were identified as still grappling with food as a means to cope and it can be a challenge to find new ways to channel emotions without turning to food [18, 19]. Participants also identified that it was easier to manage the food cravings after surgery as well as the “head hunger” which involves not having physical hunger but the desire to eat anyway.

I'm realizing now that there is a real emotional and physical effect that different foods have on your body. For example, the effect that sugar might have on your emotions or energy level, the way alcohol affects your body almost immediately post-op…these things are intensified. [P3]

I still have those gnawing cravings from time to time and I just find it easier to say you do not need it right now, you can wait. It's not like before where I had to have the food otherwise I was gonna die. [P6]

If I'm going to have something bad like sugar or something, I make sure I have protein first so I'm full, then it's not such a big deal. I try not to tell myself “no” on anything because the minute I say “no,” then I start have mental issues with it. [P5]

There are certain times where I think. Damn. I'm feeling depressed today and I wish I could just go eat a couple of pieces of cheesecake and that would only make me happy temporarily…I've tried to self-sabotage and I'm glad I get sick. It kind of conditioned me to not do that anymore…like, “hey, this is bad for you. Do not do it.” [P4]

### 3.2. Bariatric Surgery Is Not a Magic Pill

This theme was common across all participants and it deserves to be noted that many patients who passed the first two years of surgery realize (e.g., by experiencing weight regain) that bariatric surgery is intended to be used only as a “tool” to aid in weight loss or to reduce physical hunger. All participants offered words of advice and/or caution to those considering bariatric surgery.

I think that the surgery is not a fix all…it is a tool to help you achieve weight loss, but it is only a tool. It is not the end to all answers. It helps you with the amount you can eat and can definitely help you lose weight but if you still do not pick the right [food] choices to eat and exercise, it's still not going to do you any good in the long term. [P2]

Weight loss is not something that's going to happen on its own. I mean, everybody has this misconception that you have surgery and then get magically skinny and that's not how it works. You still have to put in the work and your everything into this every day. [P4]

I think people have it in their heads that you go into surgery and have it done and come out instantly thin. They do not realize that you cannot eat and drink at the same time or you can only eat 3 ounces of food at once…nobody talks about all that and all the working out and that you've been gutted like a fish, they just think you're instantly skinny within a month…I do not know why people think it's so easy…it's harder actually. It's life-long. [P7]

## 4. Discussion

Experience of participants was profiled in an attempt to share their qualities through thick description. The experience of all participants' lives after bariatric surgery provided a glimpse of what role food now plays after the first two years after operation.

Food was still considered a huge part of life, though limited in quantity, even years after bariatric surgery. Participants discussed not consuming their recommended daily intake of protein and not taking their vitamins. Low iron, B12, and vitamin D were consequences of poor supplementation. Participants compared food cravings to addictions to alcohol or drugs and there was still grief felt in regard to being unable to enjoy the same meals socially and with family as before surgery. Participants adjusted to changes in diet in different ways, and some were more successful than others, though many still fight the urges to use food to cope in certain situations. In addition, many participants experienced food intolerances and/or dumping syndrome. Many found eating a simple food such as a cracker (carbs) or any refined sugar may make them become unpleasantly ill.

Having bariatric surgery is a lifelong decision that requires follow-up care. These patients face medical and psychosocial challenges that require continuing care for years after surgery, and the hard work begins post-surgery [20]. It is also pointed out that not much has been written on the topic of follow-up care, but rather the focus has been on who is a good candidate for surgery rather than what patients go through after the procedure [20].

A qualitative study using phenomenological approach was used for this study in order to gain a better understanding of the experience of participants who were at least two years after bariatric surgery. The use of semistructured interviews allowed the participants to express their feelings and experience of life after bariatric surgery which many follow-up surveys lack and may expand the knowledge base from the participants own lived encounters with bariatric surgery. The themes around food generated from the data collected in this study provided a glimpse of life once the magic of the first year has passed and the reality of the surgery had set in. Additional emerging themes from this study to be analyzed include the role of social support and perceptions of self and society.

While obesity threatens to explode into a reverberating pandemic, there is a need to understand and support those individuals who have made the decision to have bariatric surgery. Studies such as this can offer health care professionals needed insight and information to be supportive and provide the care necessary to this vulnerable population. One participant who has struggled more than two years post-surgery with weight regain, and who stands apart from the others in current postoperative BMI (Table 3), summed up the lifelong impact of diet for the bariatric patient.

It's lifelong and a lifestyle change… I mean you cannot have your stomach cut out and then think you can eat crap food like you did before. I noticed that it is very easy to eat junk food like chips and soda…these foods go down easily…also if you drink your calories…you'll gain the weight back. There are ways to eat around bariatric surgery. I just think that if you want long-term success with bariatric surgery then it's a complete lifestyle change and it's not a temporary thing, I know that now. You cannot lose all the weight and then think you can go back to unhealthy eating like you did before surgery. You need to learn how to eat from the start of the journey.

## Figures and Tables

**Table 1:** Open-ended interview questions.

(1) How has bariatric surgery affected your quality of life now that you are 2+ years post-op?	
(2) What is different for you now than after the first year post-op?	
(3) How has bariatric surgery impacted your health? (both positively and negatively)	
(4) How has bariatric surgery impacted your emotional health?	
(5) How has bariatric surgery impacted your personal relationships?	
(6) How has weight-loss surgery (WLS) affected your nutritional intake?	
(7) What types of WLS support groups or online communities do you participate in, if any? Why or why not?	
(8) What are the things only people who have had WLS know about that most outsiders would not?	
(9) What are the cultural/social implications you have experienced since having WLS?	
(10) Tell me about your personal satisfaction with bariatric surgery.	

**Table 2:** Participant demographics.

Name	Age	Ethnicity	Marital status	Time since surgery	Surgery type
P1	54	Caucasian	Divorced	7 yrs. 5 mo.	RNYB
P2	30	Caucasian	Married	2 yrs. 11 mo.	VSG
P3	45	Hispanic	Married	2 yrs. 4 mo.	VSG
P4	27	Caucasian	Married	2 yrs. 5 mo.	VSG
P5	56	Caucasian	Married	3 yrs.	VSG
P6	57	Caucasian	Divorced	2 yrs. 7 mo.	VSG
P7	31	African-American	Single	2 yrs. 5 mo.	VSG
P8	34	Hispanic	Divorced	2 yrs. 9 mo.	RNYB
P9	45	African-American	Divorced	2 yrs. 5 mo.	VSG

**Table 3:** Self-reported participants presurgery and current anthropometrics.

Name	Day of surgery weight (pounds)	Day of surgery BMI*	Lowest weight achieved (pounds)	Postsurgery weight-loss (pounds)	Current weight (pounds)	Current BMI*
P1	289	49.6	176	113	184	31.6
P2	220	43	162	58	210	41
P3	199	36.4	139	60	139	25
P4	235	36.8	154	81	164	27.7
P5	210	38.4	140	70	145	26.5
P6	243	40.4	128	115	133	22.1
P7	298	48.1	184	114	184	29.7
P8	283	47.1	172	111	175	28.2
P9	240	39.9	170	70	170	28.3

*Note. Body Mass Index (BMI) Formula: weight (lb.)/[height (in.)]^2^ × 703.
